# Analysis of the effect of the active compound of green tea (EGCG) on the proliferation of peripheral blood mononuclear cells

**DOI:** 10.1186/1472-6882-14-322

**Published:** 2014-08-30

**Authors:** Farid Saleh, Raj Raghupathy, Sami Asfar, Medhat Oteifa, Noha Al-Saleh

**Affiliations:** Department of Anatomy, College of Medicine, Al-Imam Mohammad Ibn Saud Islamic University, Riyadh, Kingdom of Saudi Arabia; Department of Microbiology, Faculty of Medicine, Kuwait University, Kuwait City, Kuwait; Department of Surgery, Faculty of Medicine, Kuwait University, Kuwait City, Kuwait; Department of Surgery, Kuwait Cancer Control Centre, Kuwait City, Kuwait

**Keywords:** EGCG, PBMC, IFN-γ, Green tea

## Abstract

**Background:**

Cancer immunotherapy requires proper manipulation of the immune system, lymphocytes in particular, in order to identify and destroy the cancer cells as non-self. In this study we investigated the effect of the flavonoid present in green tea, namely epigallocatechin-3-gallate (EGCG), on the proliferation of, and IFN-γ production by, peripheral blood mononuclear cells (PBMC) from breast cancer patients stimulated with a mitogen, anti-CD3 and the common breast cancer peptides Her-2/neu, and p53.

**Methods:**

Blood samples were collected from 25 patients with breast cancer at the Kuwait Cancer Control Centre (KCCC). The patients were newly diagnosed, and had not undergone any treatment or surgery at the time of sample collection. The control group consisted of 25 healthy women age-matched (±5 years) to the patients. PBMC were isolated from the patients and controls, and were cultured separately with the mitogen PHA, anti-CD3 antibodies, and Her-2/neu and p53 in the presence or absence of standardized doses of EGCG. The degree of proliferation and interferon-γ [IFN-γ) release were then analyzed.

**Results:**

EGCG significantly suppressed the proliferation of PBMC in response to stimulation separately with (i) the mitogen, (ii) anti-CD3, and (iii) the cancer antigen peptides. IFN-γ production was also significantly suppressed by EGCG in vitro.

**Conclusions:**

EGCG appears to have an immunosuppressive effect on the proliferation of PBMC, indicating that EGCG is worth exploring for immunomodulatory effects in autoimmune diseases and tissue transplantation.

## Background

Green tea contains polyphenols, particularly catechins, the most abundant of which is Epigallocatechin-3-gallate (EGCG) [1–4]. It has been reported that EGCG can exert in vitro antitumor effects in various types of cancer, such as cancer of the breast, esophagus, prostate, skin, stomach, lung, colon, and pancreas [1, 5–7]. Studies have shown that EGCG mediates its anti-cancer effect by regulating cancer cell angiogenesis and metastasis [1, 2, 8]. Another important characteristic of EGCG is its relatively low systemic toxicity [9, 10]. Thus, EGCG has been proposed to merit possible use as an adjuvant or immunostimulant in cancer therapy [11].

Several investigations have highlighted the possible role of the immune system in cancer treatment. These include studies which showed a positive correlation between tumor-infiltrating T lymphocytes and patient survival, and where tumor-specific T cell responses were found in individuals with premalignant lesions and in patients with recurrent primary tumors [12–20]. Cytotoxic T lymphocytes (CTLs) isolated from some of these patients, cultured in vitro with whole tumor lysate or with dendritic cells (DCs) pulsed with tumor antigens/peptides, and then returned to the cancer patients were found to be capable of targeting the tumor causing its partial or complete regression [21]. An important principle in cancer immunotherapy is to get CD8^+^ CTLs to recognize tumors as foreign, and to recruit them to the tumor site. This generally requires help from T helper lymphocytes (CD4^+^ T cells), and from antigen-presenting cells (APCs) like the DCs.

Studies investigating the effect of EGCG on the immune system have yielded varying results. Kang and colleagues reported that combining HPV DNA vaccine with oral EGCG intake resulted in an enhanced tumor-specific T-cell immune response, and an enhanced antitumor effect in mice injected with three tumor cell lines (TC-1, B16, and B16E7) [22]. This also resulted in a high cure rate, with a long-term antitumor protection in the cured mice. In fact, the cured animals were capable of rejecting a challenge with E7-expressing tumors like TC-1 and B16E7 weeks after the combined therapy, and they showed significant antigen-specific immune responses [22]. On the other hand, Kawai and co-workers reported that EGCG exerted down-regulation of CD11b expression on CD8^+^ T cells [23]. Being a member of the α-chain integrin family, CD11b can form a complex with β2-integrin known as Mac-1. CD11b is often expressed on monocytes, neutrophils, a subset of CD8+ T cells, and natural killer cells, and has a key role in helping lymphocytes migrate from the intravascular space into the target tissues [24, 25]. Accordingly, the presence of circulating and interstitial CD8+CD11b+ T cells represents recently-activated effector cells [26, 27].

Given the fact that the effect of EGCG on lymphocytes is still open to further investigation, we sought to observe the effect of EGCG on the proliferation of, and IFN-γ production by, peripheral blood mononuclear cells from newly-diagnosed breast cancer patients.

## Methods

### Ethics approval

The study was approved by the Ethics Committees of the Faculty of Medicine, Kuwait University. Both patients and controls gave informed consent after receiving a detailed description of the study.

### Blood samples

Six milliliters of venous blood was collected in heparinized tubes from both patients and controls. Blood samples were transported directly to the Cellular Immunology Laboratory at the Faculty of Medicine, Kuwait University. Separation of peripheral blood mononuclear cells (PBMC) and stimulation of cells were performed within two hours of withdrawal of blood.

### Procedure

PBMC were separated by Ficoll-hypaque density gradient centrifugation, suspended in RPMI medium containing 10% fetal calf serum, aliquoted into 96 well tissue culture plates at a density of 10^5^ cells per well, and then challenged with the breast cancer antigen peptides (p53: sequence: TYSPALNKMF; Her-2/neu: sequence: KIFGSLAFL; JPT Peptide Technologies, Germany) for six days, or with 5 μg/ml of the mitogen phytohemagglutinin (PHA) (Sigma Aldrich, St. Louis, USA) for a period of four days. For stimulation with anti-CD3 antibodies, tissue culture plates were first coated overnight with anti-CD3 antibodies (Bender MedSystems, Vienna, Austria) followed by washing with sterile phosphate buffered saline (PBS). PBMC were then aliquoted into the wells of the plates. Cell proliferation was measured by estimating the incorporation of ^3^H-thymidine (GE Healthcare, Uppsala, Sweden). Culture supernatants were collected 4 days following activation with mitogen or anti-CD3 and after 6 days following activation with breast cancer antigen peptides in order to measure the levels of IFN-γ by ELISA. PBMC were treated in triplicates as shown below:

1. PBMC + tissue culture medium alone (negative control).
2. PBMC + Mitogen or Antigen or anti-CD3 alone (positive control).
3. PBMC + Antigen or Mitogen or anti-CD3 + EGCG.

### ELISA for the estimation of IFN-γ

The manufacturer’s protocols (Beckman Coulter, Marseilles, France) were followed for these assays, which are based on the antibody sandwich principle; samples and standards were first incubated with a solid phase monoclonal antibody which captures the specific cytokine present. The solid phase-bound cytokine was then incubated with a second antibody such as biotinylated anti-cytokine antibody. The resulting antigen-antibody complexes were then incubated with a streptavidin-enzyme conjugate after which the substrate and chromogen were added. The resulting color development, which is directly proportional to the amount of the cytokine present in the sample and standards, was stopped after 5 minutes. Samples were tested in triplicate, and the absorbance values read using an ELISA Reader. Accurate sample concentrations of cytokines were determined by comparing their respective absorbencies with those obtained for the reference standards plotted on a standard curve.

### Statistical analysis

Data were analyzed using ANOVA (SPSS statistical package for Windows). All data are expressed as mean ± Standard Error of the mean (SEM). A difference was considered significant if the P value was ≤ 0.05.

## Results

### Initial standardization of optimal EGCG concentration

PBMC were stimulated with PHA or anti-CD3 or the breast cancer peptides in the presence and absence of EGCG for four days after which the cells were incubated with radioactive thymidine for 18 hours; cells were then harvested and the radioactivity estimated. In order to find the optimal concentration of EGCG, it was tested at seven concentrations 50 μg/ml, 10 μg/ml, 5 μg/ml, 1 μg/ml, 0.5 μg/ml, 0.25 μg/ml and 0.125 μg/ml. PBMC from five breast cancer patients were exposed to the mitogen in the absence and presence of EGCG. A significant suppression of PBMC was observed with an EGCG concentration of 50 μg/ml (p < 0.0001), and to a lesser extent with 10 μg/ml (p < 0.01). Lower concentrations of EGCG did not show any suppression.

PBMC were incubated either with anti-CD3 alone, or with anti-CD3 along with different concentrations of EGCG. The results showed that an EGCG concentration of 50 μg/ml induced a significant suppression of PBMC proliferation (p < 0.0001), which was also observed at an EGCG concentration of 10 μg/ml (p < 0.01). No significant suppression of proliferation was observed at lower EGCG concentrations.

The two breast cancer antigen peptides Her-2/neu and p53 were tested at five different concentrations, namely 5 μg/ml, 10 μg/ml, 20 μg/ml, 40 μg/ml, and 80 μg/ml. EGCG suppressed the proliferation of PBMC in response to both breast cancer antigen peptides; this suppression was stronger when EGCG was tested at 50 μg/ml (p < 0.0001), and to a lesser extent when tested at 10 μg/ml (p < 0.01). No suppression was seen when EGCG was used at lower concentrations. Accordingly, we proceeded to test the samples obtained from the 25 breast cancer patients and 23 healthy age-matched controls using two concentrations of EGCG, namely 50 μg/ml and 10 μg/ml. The data presented here pertain to the EGCG 50 μg/ml and experiments. Data pertaining to the EGCG 10 μg/ml experiments are not presented, but will be provided upon request. Also, we report here results pertaining to the peptide concentration of 80 μg/ml experiments. Results pertaining to the peptide concentration of 40 μg/ml will be provided upon request.

### Effect of EGCG on mitogen-induced proliferation and IFN-γ release

As shown in Table 1, a significant suppression of proliferation of PBMC against PHA (shown as cpm of thymidine uptake) was detected in the presence of an EGCG concentration of 50 μg/ml (p = 0.0001). The stimulation index calculated as counts per minute of test culture divided by counts per minute of control culture was also significant (p = 0.0001). EGCG at a concentration of 50 μg/ml also suppressed IFN-γ production (p = 0.003). No significant differences in stimulation indices and IFN-γ production were observed between breast cancer patients and normal healthy controls.

**Table 1:** **The effect of epigallocatechin-3-gallate (EGCG) on breast cancer peripheral blood mononuclear cells (PBMC) proliferation and IFN-γ production**

^3^H thymidine uptake by PBMC (cpm ± SEM)			Stimulation indices [SI] of PBMCs) (SI ± SEM)		IFN-γ release by PBMCs (pg/ml ± SEM)		
Cell control	Mitogen alone	Mitogen + EGCG	Mitogen alone	Mitogen + EGCG	Cell control	Mitogen alone	Mitogen + EGCG
69 ± 7	11289 ± 1637	33 ± 4	172 ± 26	0.4 ± 0.04^§^	53 ± 8	2153 ± 454	38 ± 12^§^
**Cell control**	**Anti-CD3 alone**	**Anti-CD3 + EGCG**	**Anti-CD3 alone**	**Anti-CD3 + EGCG**	**Cell control**	**Mitogen alone**	**Anti-CD3 + EGCG**
199 ± 29	39252 ± 4182	73 ± 14	262 ± 49	1 ± 0.05	54 ± 42	24508 ± 717	10 ± 4
**Cell control**	**Her-2/neu alone**	**Her-2/neu + EGCG**	**Her-2/neu alone**	**Her-2/neu + EGCG**	**Cell control**	**Her-2/neu alone**	**Her-2/neu + EGCG**
101 ± 8	432 ± 79	40 ± 6	4.3 ± 0.7	0.4 ± 0.07	13 ± 6	62 ± 12	28 ± 9
**Cell control**	**p53 alone**	**p53 + EGCG**	**p53 alone**	**p53 + EGCG**	**Cell control**	**p53 alone**	**p53 + EGCG**
72 ± 9	972 ± 191	39 ± 5	9 ± 1	0.4 ± 0.06	52 ± 8	109 ± 11	33 ± 5

*cpm*: Counts per minute; *SEM*: Standard error from the mean; Mitogen: phytohemagglutinin (PHA); EGCG (50 μg/ml); *IFN-γ*: Interferon gamma; *pg/ml*: Picogram per ml. Her-2/neu and p53 (80 μg/ml). All results obtained by EGCG were significant at p < 0.01, as indicated by the symbol “§”.

### Effect of EGCG on anti-CD3-induced proliferation and IFN-γ release

The results obtained with anti-CD3 were similar to those seen with PHA. As shown in Table 1, EGCG significantly suppressed the proliferation of PBMC against anti-CD3 (p = 0.003), as measured by thymidine uptake, when it was added to the culture at a concentration of 50 μg/ml. Similar observations were made with stimulation indices (p = 0.003), and IFN-γ production (p = 0.003). No significant differences were observed in the extent of suppression between cancer patients and healthy controls.

### Effect of EGCG on Her-2/neu-induced proliferation and IFN-γ release

The effect of EGCG on breast cancer antigen peptide-induced proliferation was tested in the 25 breast cancer patients. EGCG significantly suppressed Her-2/neu (80 μg/ml)-peptide-induced proliferation (thymidine uptake), when added at a concentration of 50 μg/ml (p = 0.002) (Table 1). A similar trend was observed when the peptide concentration was 40 μg/ml (p = 0.002).

Stimulation indices showed significant (p =0.002) suppression similar to those seen with thymidine uptake, when EGCG was used at a concentration of 50 μg/ml in the presence of 80 μg/ml of the Her-2/neu antigen peptide (Table 1). Again, a similar trend was observed when the peptide concentration was 40 μg/ml (p = 0.002).

As far as production of IFN-γ by stimulated PBMC is concerned, EGCG induced significant suppression at a concentration of 50 μg/ml in the presence of 80 μg/ml (p = 0.04) and 40 μg/ml (p = 0.05) of the Her-2/neu antigen peptide (Table 1).

### Effect of EGCG on p53-induced proliferation and IFN-γ release

As shown in Table 1, significant suppression was seen with the p53 antigen peptide. Proliferation of PBMC induced with 80 μg/ml of the peptide was significantly suppressed when EGCG was added at 50 μg/ml (p = 0.002). Likewise, EGCG suppressed the proliferation of PBMC (p = 0.002), when PBMC were stimulated with 40 μg/ml of the peptide.

Stimulation indices showed similar suppression; PBMC stimulated with p53 at 80 μg/ml were suppressed when EGCG was added at 50 μg/ml (p = 0.002) (Table 1). Similarly, PBMC stimulated with 40 μg/ml of the p53 antigen peptide were significantly suppressed when EGCG was added at 50 μg/ml (p = 0.002).

Finally, the production of IFN-γ by PBMC stimulated with 80 μg/ml of the p53 peptide antigen was significantly suppressed when EGCG was added at 50 μg/ml (p = 0.009) (Table 1). EGCG also significantly suppressed IFN-γ production (p = 0.05) in the presence of the p53 peptide antigen at a concentration of 40 μg/ml.

### Effect of EGCG on cell viability

To ascertain whether EGCG brings about an inhibition of cell proliferation by killing peripheral blood cells, we measured cell viability in nine samples at random using the Trypan Blue Dye Exclusion Test. This test is based on the principle that viable cells do not allow uptake of the dye, while the dye enters dead cells which then appear blue under the microscope. We measured the viability of cells cultured with medium alone, with EGCG, and with PHA plus EGCG at concentrations ranging from 1 μg/ml to 50 μg/ml. Cell viability in cultures with medium alone was 99-100%, and viability in cultures with EGCG was 98-100%. Cells in cultures with PHA alone had a viability of 95-98%, while those in cultures with PHA and EGCG at different concentrations also had a viability ranging from 95 to 98%. These experiments indicate that EGCG, at least at the concentrations tested here, does not affect cell viability. Therefore, we suggest that the decrease in cell proliferation and IFN-γ production is related to suppression rather than to a cytotoxic effect.

## Discussion

The benefit of the antioxidant component of green tea on human health, especially in preventing cancer development, has been the subject of investigation over the last decade. Drinking green tea has been reported to significantly decrease the risk of developing gastrointestinal, breast, epithelial ovarian, and prostate cancer, to name a few conditions [28–33]. In addition to cancer prevention, the therapeutic usage of green tea in treating cancer has also been studied, and has yielded promising results in prostate, breast, and lung cancer, as well in chronic lymphocytic leukemia [34].

Cancer immunotherapy relies on stimulating the immune system of the patient against cancer cell peptide antigens. For this to take place, immune cells must go through a cascade of events involving antigen recognition, presentation, and destruction of the cancer cells expressing these antigens. The immune cells must also prevent the attempts of the cancer cells to evade their surveillance. In this study, we investigated the possibility of stimulating the proliferation of PBMC isolated from breast cancer patients against two common breast cancer antigens Her-2/neu and p53, using EGCG in culture. The most abundant catechin in green tea is EGCG, which represents around 50% of catechins found in green tea. Therefore, any effect of green tea extract is attributed to this catechin. We based our investigation on the demonstration that consumption of green tea has already been shown to yield positive results in certain types of human cancer, as described earlier. Therefore, if EGCG is capable of enhancing the proliferation of PBMC, this could be further investigated in relation to using it as part of peptide-based vaccine immunotherapy of cancer. Surprisingly, our results showed that EGCG induced suppression of proliferation (measured by both thymidine uptake and stimulation index) of PBMC when used at a standardized dose of 50 μg/ml and 10 μg/ml. No significant suppression was observed at doses lower than 10 μg/ml. Such suppression was also observed in the amount of IFN-γ released in culture. We have also demonstrated that that the results obtained in this study were due to suppression of proliferation of PBMC and not due to cytotoxicity exerted by EGCG.

Our results are in accord with those reported by Wu and colleagues, who investigated the effect of EGCG on immune cells isolated from mice [35]. The authors reported that proliferation of T cells was inhibited by EGCG at physiologically relevant concentrations. EGCG inhibited T cell division and cell cycle progression in a dose-dependent manner, and no cytotoxicity or apoptosis were observed. Moreover, the authors reported that the presence of EGCG resulted in higher IL-2 accumulation and lower IL-2 receptor expression, thus implying an impeded IL-2/IL-2 receptor signaling [35]. Other studies have also demonstrated the suppressive effect of EGCG on the immune system. Aktas and co-workers reported that EGCG administered orally to mice significantly abrogated proliferation and TNF-α production of T cells [36]. The authors also reported the induction of CD4^+^ T cell cycle arrest resulting in down-regulation of cyclin-dependent kinase 4. Further analysis revealed that the interference with T cell growth and function was mediated by the blockade of the catalytic activities of the 20S/26S proteasome complex, resulting in intracellular accumulation of I_*k*_B-α and subsequent inhibition of NF_*k*_B activation [36]. Hu and colleagues demonstrated that EGCG significantly inhibited the proliferation of B and T cells in culture [37]. Similarly, Wilasrusmee and co-workers and Zvetkova and colleagues reported that green tea extract inhibited the proliferation of murine lymphocytes in vitro, and the production of neopterin, a marker for cellular immunity by human PBMC, respectively [38, 39]. Other studies have demonstrated suppression of maturation of mouse bone marrow-derived dendritic cells (DCs), and antigen-presenting cells derived from human monocytes when cultured with EGCG [40, 41]. T cells co-cultured with EGCG-treated DCs also exhibited decreased proliferation EGCG [40, 41]. In a recent study conducted by Pae and colleagues, the authors confirmed a direct inhibitory effect of EGCG on T cells, and that this was more pronounced in CD4^+^ T cells, as compared to CD8^+^ T cells [42]. EGCG also inhibited accessory and antigen-presenting cell function, which further strengthened its inhibition of T cells. More recent studies have further demonstrated the suppressive role of EGCG on the various arms of the immune system in cancer cases [43–49]. In a study conducted by Mukherjee and colleagues, the authors reported that treatment with EGCG resulted in the inhibition of cytokine and chemokine gene induction, cell migration, and activity of MMP-9 and -2 in prostate cancer [43]. Ren and co-workers demonstrated a dose-dependent suppression of migration of lipopolysaccharide (LPS)-activated macrophagic RAW264.7 cells [44]. Chen and colleagues reported that some anti-inflammatory phytochemicals like EGCG appear to modulate the tumor microenvironment by repressing NF-kappaB (NF-κB, a proinflammatory transcription factor), and inhibiting pro-inflammatory cytokines like IL-6 and TNF-α in epithelial ovarian cancer [45]. In a study conducted on melanoma, Ellis and co-workers demonstrated a significant reduction in the levels of cyclooxygenase (COX)-2, prostaglandin (PG) E(2), and PGE(2) receptors (EP2 and EP4) when EGCG was administered [46]. Similar results were reported by Singh and Kativar [47]. Hoffmann and colleagues reported both dose-dependent and time-dependent down-regulation of IL-1 (a key player in inflammation-associated cancer development) by EGCG in pancreatic cancer [48]. Kürbitz and co-workers reported that green tea catechins like epicatechin gallate and catechin gallate were superior to EGCG in inhibiting TNFα-induced activation of NF-κB and consequently secretion of pro-inflammatory and invasion promoting proteins like uPA and IL-8, when cultured with pancreatic tumor cells [49].

## Conclusion

Our results demonstrate that EGCG has an inhibitory effect on the proliferation of PBMC as well as the production of IFN-γ by PBMC isolated from breast cancer patients when cultured with the breast cancer peptide antigens Her-2/neu and p53 as well as with the mitogen PHA and with anti-CD3 antibodies. Although such observations may contradict the use of EGCG in cancer immunotherapy, it may open the door to further studies on its use as an immunomodulatory agent in autoimmune diseases and tissue transplantation.
